# 

**Published:** 1889-03

**Authors:** 


CONTENTS

Vol. 36 No. 3.

| | PAGE. |
|---|---|
| The Raid of the M. D'S | 49 |
| A Victim of Trances | 50 |
| Science of Psychometry | 51 |
| San Marino | 55 |
| Magnetic Hygiene | 56 |
| Monaco | 58 |
| Stick to it, Brethren | 59 |
| A Materialist Converted | 60 |
| Domestic Science | 61 |
| Concerning Criminals | 62 |
| The Human Body as a Machine | 65 |
| The Camphor Tree | 67 |
| Book Notices | 68 |
| Household | 69 |
| Miscellaneous | 71 |
| In Memoriam | 72 |

INDEX TO ADVERTISEMENTS OF HALF A PAGE AND OVER.

| | PAGE. |
|---|---|
| Murdock's Liquid Food Co | I |
| Esoteric Publishing Co | II |
| Zylonite | II |
| The Lost Atlantis | III |
| Lactopeptine | V |
| Female Farmers | VI |
| Revised Premium List | VIII |
| Rio Chemical Co | X |
| The Herb Cure Co | XI |

